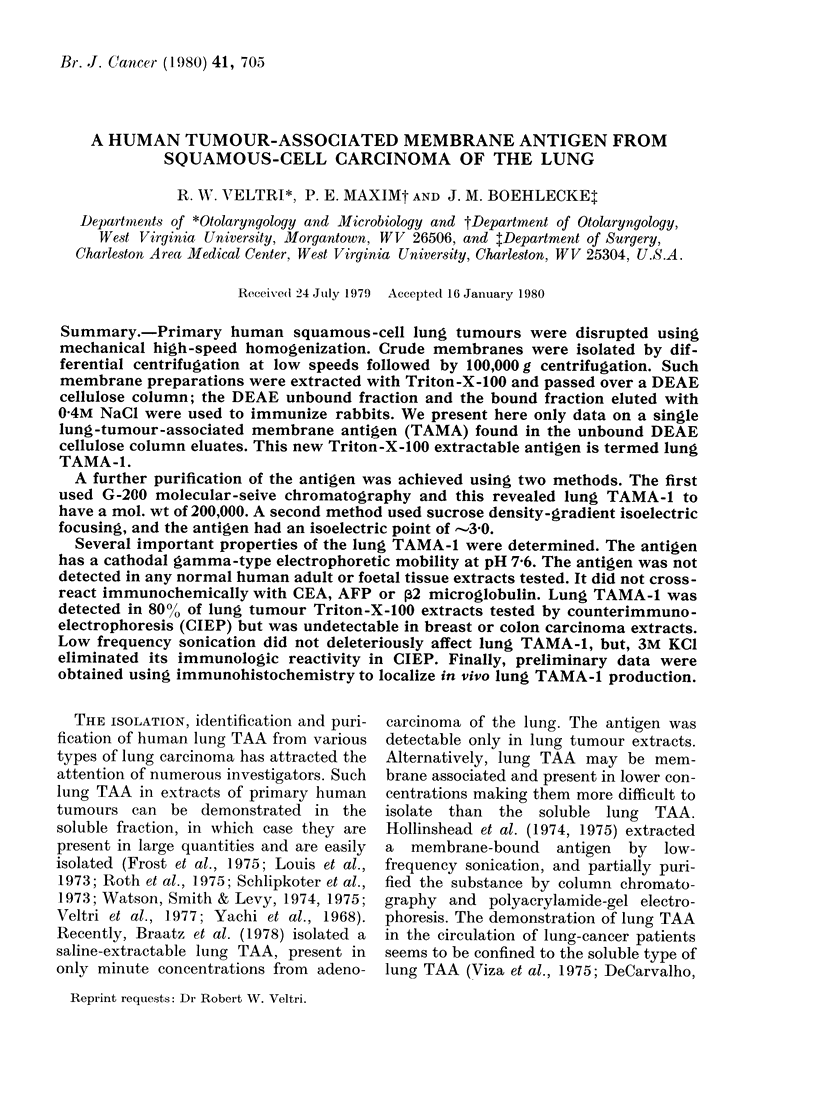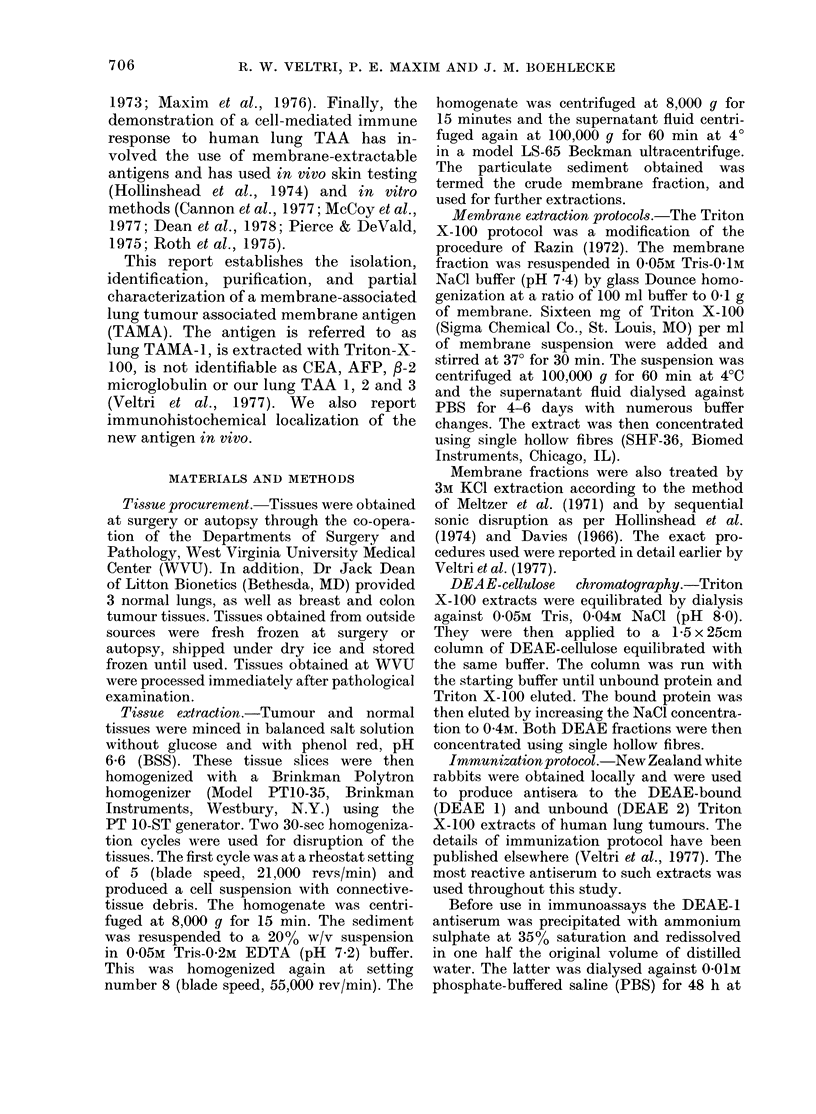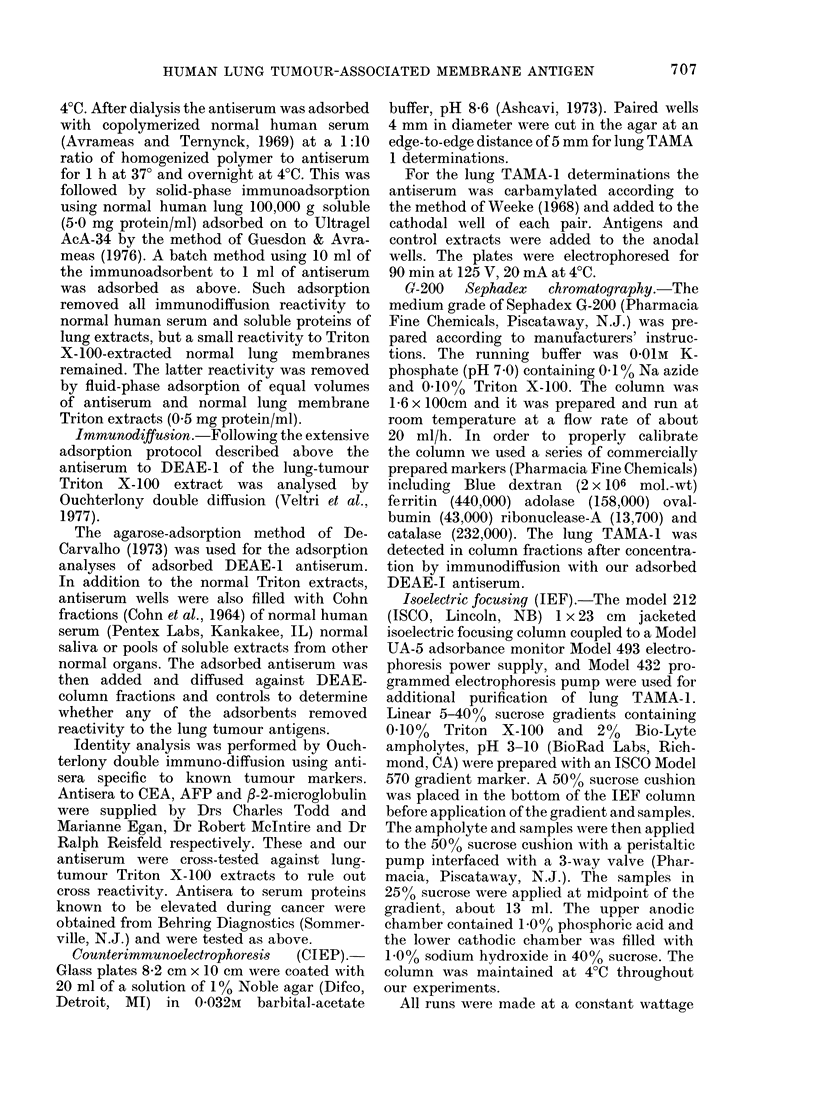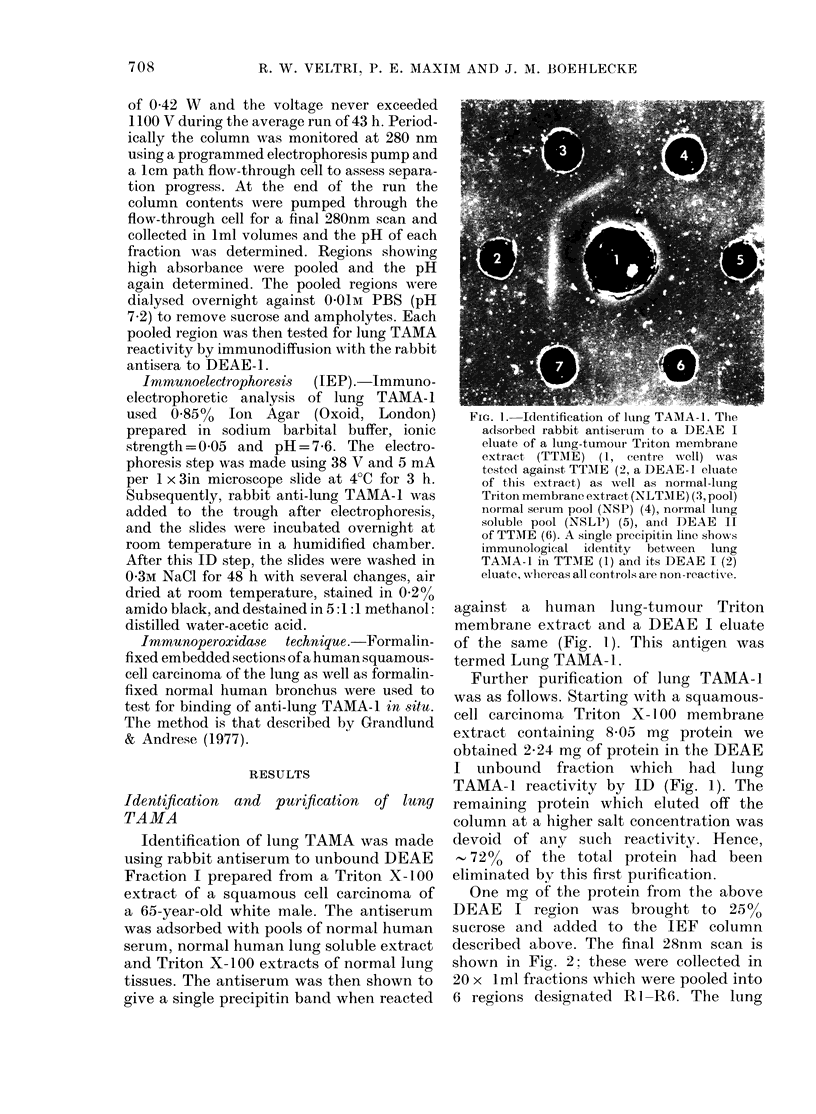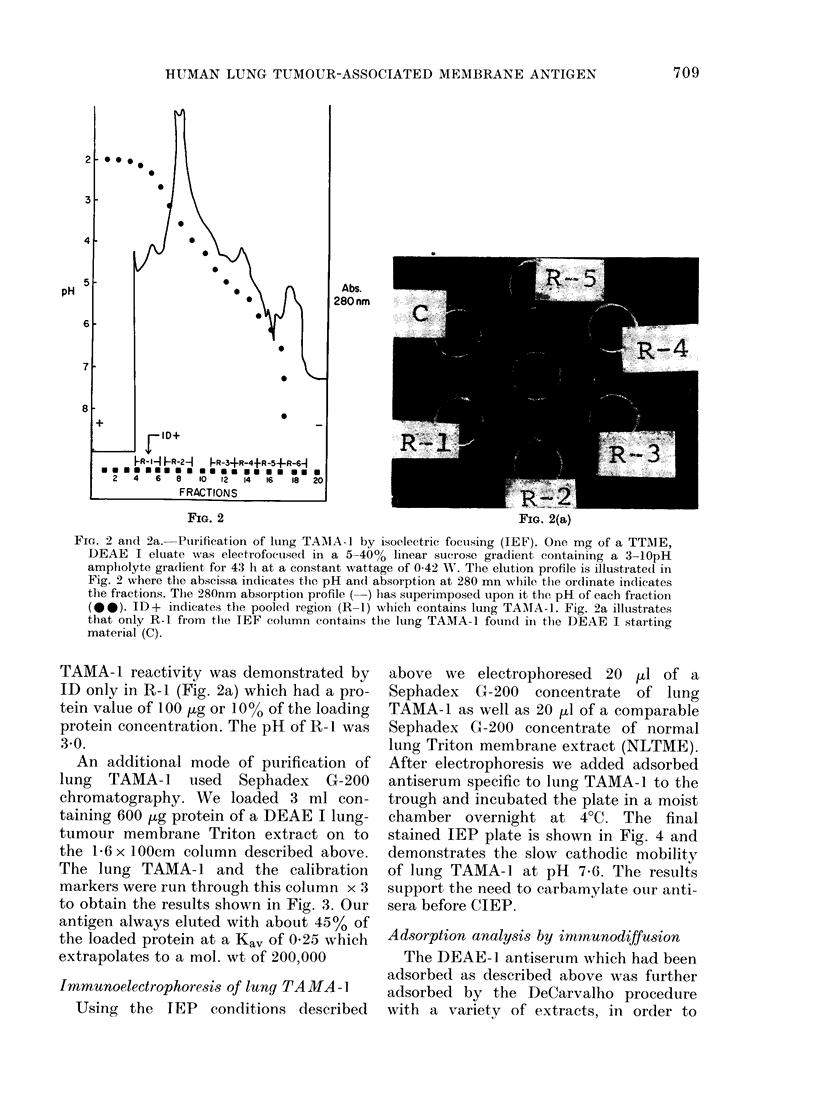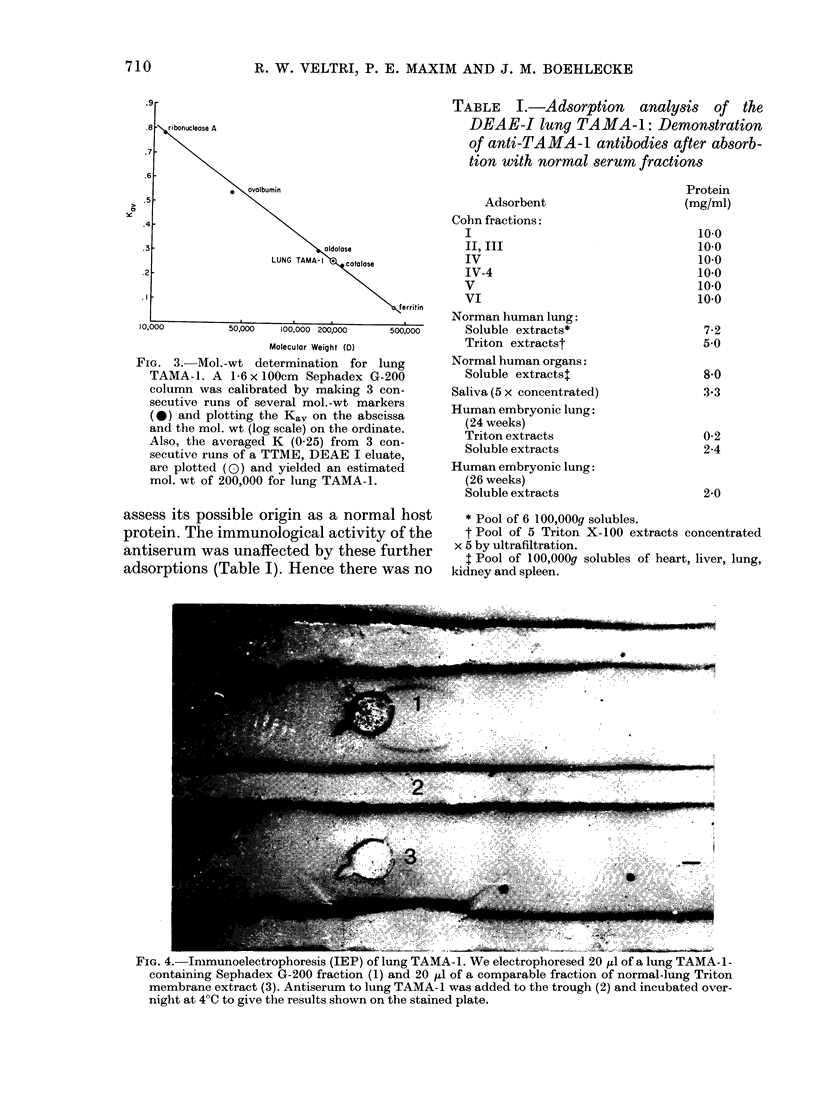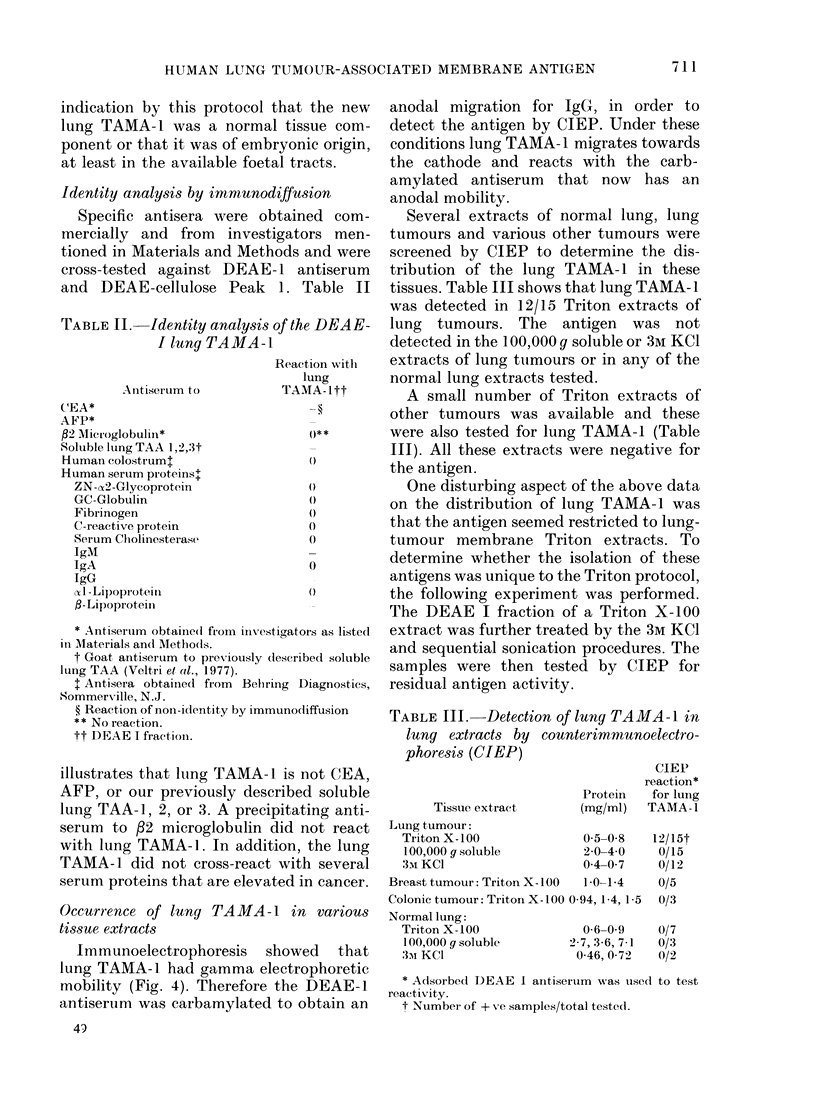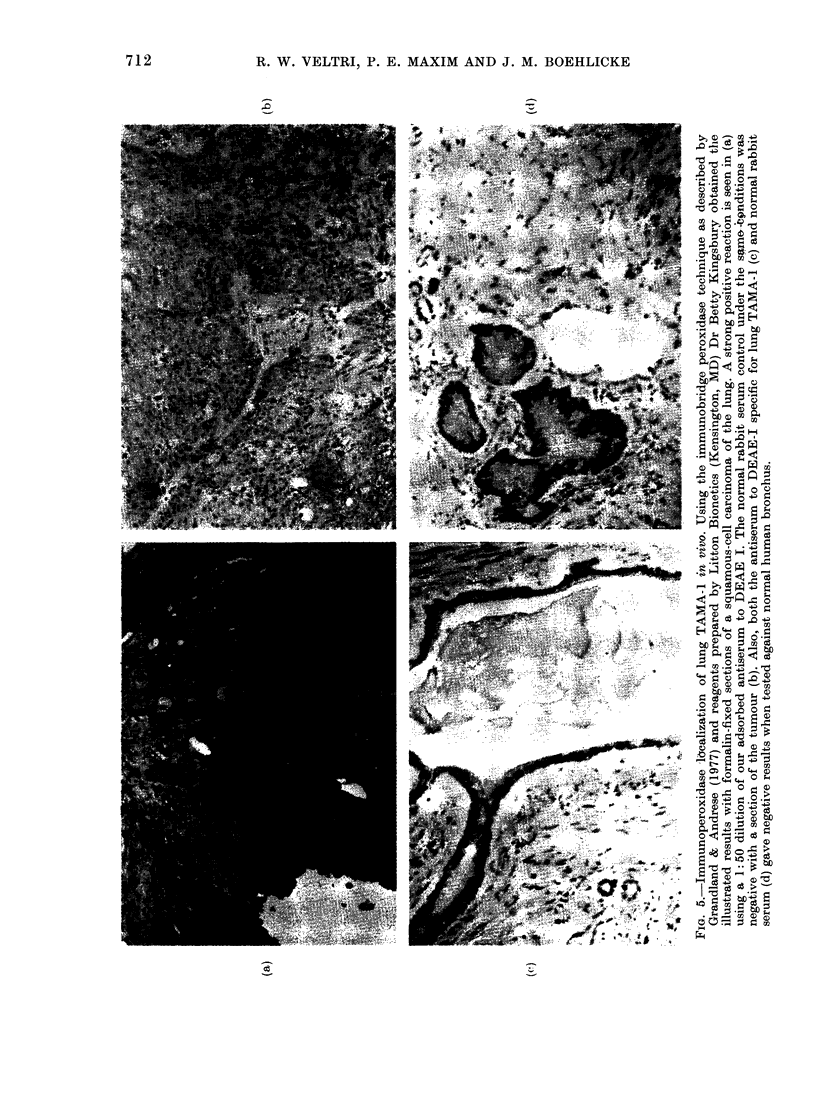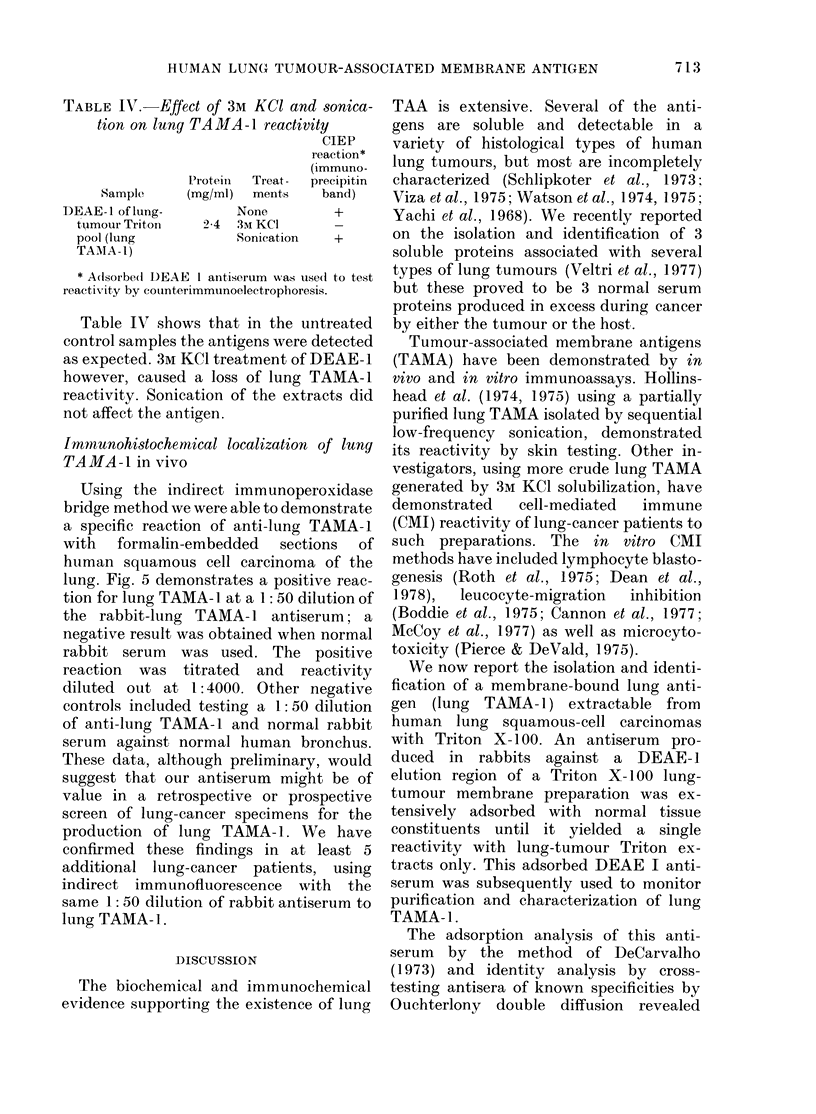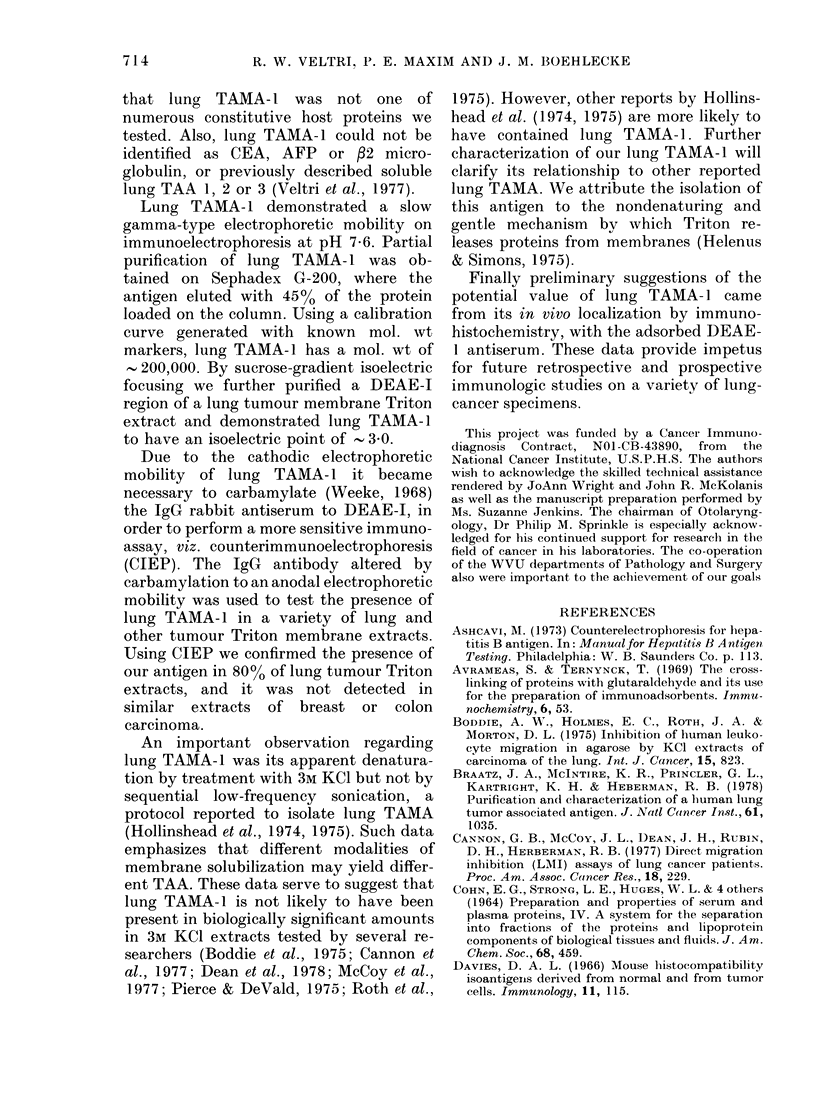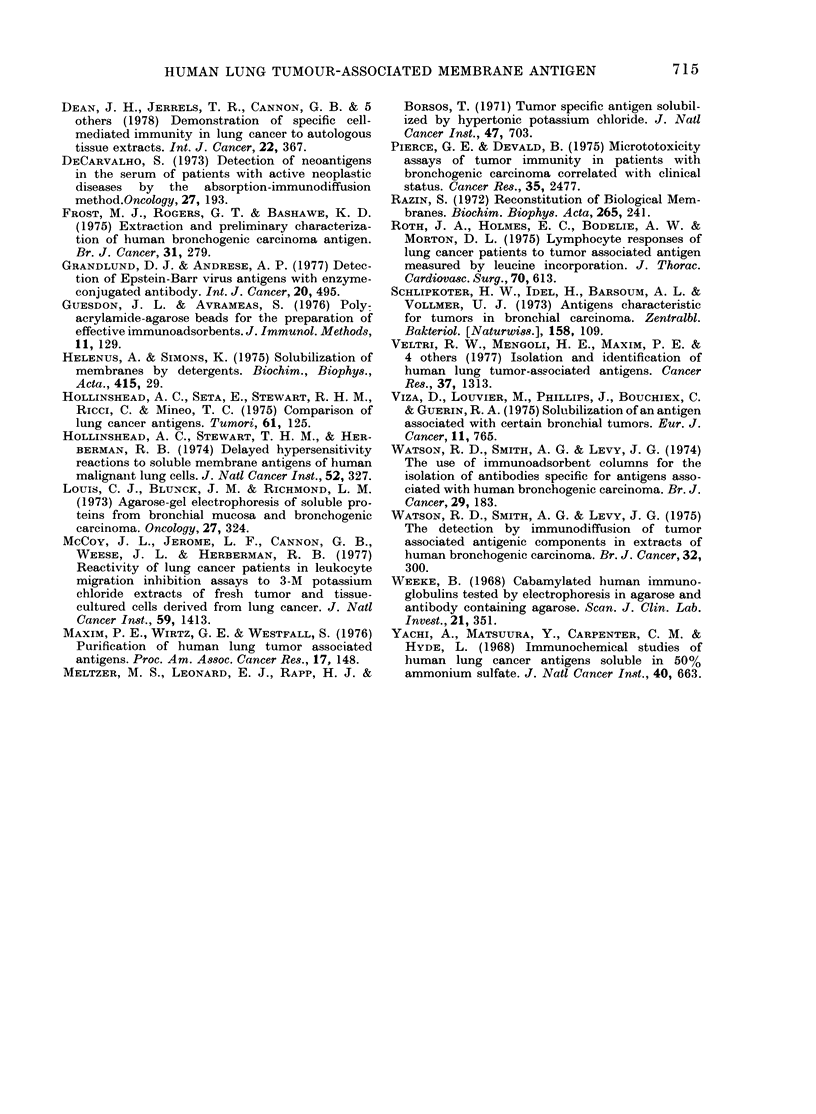# A human tumour-associated membrane antigen from squamous-cell carcinoma of the lung

**DOI:** 10.1038/bjc.1980.132

**Published:** 1980-05

**Authors:** R. W. Veltri, P. E. Maxim, J. M. Boehlecke

## Abstract

**Images:**


Br. J. Cancer (1980) 41, 705

### A HUMAN TUMOUR-ASSOCIATED MEMBRANE ANTIGEN FROM SQUAMOUS-CELL CARCINOMA OF THE LUNG

R. W. VELTRI*, P. E. MAXIM† AND J. M. BOEHLECKE‡

*Departments of *Otolaryngology and Microbiology and †Department of Otolaryngology, West Virginia University, Morgantown, WV 26506, and ‡Department of Surgery, Charleston Area Medical Center, West Virginia University, Charleston, WV 25304, U.S.A.*

Received 24 July 1979 Accepted 16 January 1980

**Summary.—Primary human squamous-cell lung tumours were disrupted using mechanical high-speed homogenization. Crude membranes were isolated by differential centrifugation at low speeds followed by 100,000 *g* centrifugation. Such membrane preparations were extracted with Triton-X-100 and passed over a DEAE cellulose column; the DEAE unbound fraction and the bound fraction eluted with 0·4M NaCl were used to immunize rabbits. We present here only data on a single lung-tumour-associated membrane antigen (TAMA) found in the unbound DEAE cellulose column eluates. This new Triton-X-100 extractable antigen is termed lung TAMA-1.**

**A further purification of the antigen was achieved using two methods. The first used G-200 molecular-seive chromatography and this revealed lung TAMA-1 to have a mol. wt of 200,000. A second method used sucrose density-gradient isoelectric focusing, and the antigen had an isoelectric point of ~3·0.**

**Several important properties of the lung TAMA-1 were determined. The antigen has a cathodal gamma-type electrophoretic mobility at pH 7·6. The antigen was not detected in any normal human adult or foetal tissue extracts tested. It did not cross-react immunochemically with CEA, AFP or β2 microglobulin. Lung TAMA-1 was detected in 80% of lung tumour Triton-X-100 extracts tested by counterimmuno-electrophoresis (CIEP) but was undetectable in breast or colon carcinoma extracts. Low frequency sonication did not deleteriously affect lung TAMA-1, but, 3M KCl eliminated its immunologic reactivity in CIEP. Finally, preliminary data were obtained using immunohistochemistry to localize *in vivo* lung TAMA-1 production.**

THE ISOLATION, identification and purification of human lung TAA from various types of lung carcinoma has attracted the attention of numerous investigators. Such lung TAA in extracts of primary human tumours can be demonstrated in the soluble fraction, in which case they are present in large quantities and are easily isolated (Frost *et al.*, 1975; Louis *et al.*, 1973; Roth *et al.*, 1975; Schlipkoter *et al.*, 1973; Watson, Smith & Levy, 1974, 1975; Veltri *et al.*, 1977; Yachi *et al.*, 1968). Recently, Braatz *et al.* (1978) isolated a saline-extractable lung TAA, present in only minute concentrations from adeno-carcinoma of the lung. The antigen was detectable only in lung tumour extracts. Alternatively, lung TAA may be membrane associated and present in lower concentrations making them more difficult to isolate than the soluble lung TAA. Hollinshead *et al.* (1974, 1975) extracted a membrane-bound antigen by low-frequency sonication, and partially purified the substance by column chromatography and polyacrylamide-gel electrophoresis. The demonstration of lung TAA in the circulation of lung-cancer patients seems to be confined to the soluble type of lung TAA (Viza *et al.*, 1975; DeCarvalho,

Reprint requests: Dr Robert W. Veltri.

1973; Maxim *et al.*, 1976). Finally, the demonstration of a cell-mediated immune response to human lung TAA has involved the use of membrane-extractable antigens and has used *in vivo* skin testing (Hollinshead *et al.*, 1974) and *in vitro* methods (Cannon *et al.*, 1977; McCoy *et al.*, 1977; Dean *et al.*, 1978; Pierce & DeVald, 1975; Roth *et al.*, 1975).

This report establishes the isolation, identification, purification, and partial characterization of a membrane-associated lung tumour associated membrane antigen (TAMA). The antigen is referred to as lung TAMA-1, is extracted with Triton-X-100, is not identifiable as CEA, AFP, β-2 microglobulin or our lung TAA 1, 2 and 3 (Veltri *et al.*, 1977). We also report immunohistochemical localization of the new antigen *in vivo*.

### MATERIALS AND METHODS

*Tissue procurement.*—Tissues were obtained at surgery or autopsy through the co-operation of the Departments of Surgery and Pathology, West Virginia University Medical Center (WVU). In addition, Dr Jack Dean of Litton Bionetics (Bethesda, MD) provided 3 normal lungs, as well as breast and colon tumour tissues. Tissues obtained from outside sources were fresh frozen at surgery or autopsy, shipped under dry ice and stored frozen until used. Tissues obtained at WVU were processed immediately after pathological examination.

*Tissue extraction.*—Tumour and normal tissues were minced in balanced salt solution without glucose and with phenol red, pH 6·6 (BSS). These tissue slices were then homogenized with a Brinkman Polytron homogenizer (Model PT10-35, Brinkman Instruments, Westbury, N.Y.) using the PT 10-ST generator. Two 30-sec homogenization cycles were used for disruption of the tissues. The first cycle was at a rheostat setting of 5 (blade speed, 21,000 revs/min) and produced a cell suspension with connective-tissue debris. The homogenate was centrifuged at 8,000 *g* for 15 min. The sediment was resuspended to a 20% w/v suspension in 0·05M Tris-0·2M EDTA (pH 7·2) buffer. This was homogenized again at setting number 8 (blade speed, 55,000 rev/min). The homogenate was centrifuged at 8,000 *g* for 15 minutes and the supernatant fluid centrifuged again at 100,000 *g* for 60 min at 4° in a model LS-65 Beckman ultracentrifuge. The particulate sediment obtained was termed the crude membrane fraction, and used for further extractions.

*Membrane extraction protocols.*—The Triton X-100 protocol was a modification of the procedure of Razin (1972). The membrane fraction was resuspended in 0·05M Tris-0·1M NaCl buffer (pH 7·4) by glass Dounce homogenization at a ratio of 100 ml buffer to 0·1 g of membrane. Sixteen mg of Triton X-100 (Sigma Chemical Co., St. Louis, MO) per ml of membrane suspension were added and stirred at 37° for 30 min. The suspension was centrifuged at 100,000 *g* for 60 min at 4°C and the supernatant fluid dialysed against PBS for 4–6 days with numerous buffer changes. The extract was then concentrated using single hollow fibres (SHF-36, Biomed Instruments, Chicago, IL).

Membrane fractions were also treated by 3M KCl extraction according to the method of Meltzer *et al.* (1971) and by sequential sonic disruption as per Hollinshead *et al.* (1974) and Davies (1966). The exact procedures used were reported in detail earlier by Veltri *et al.* (1977).

*DEAE-cellulose chromatography.*—Triton X-100 extracts were equilibrated by dialysis against 0·05M Tris, 0·04M NaCl (pH 8·0). They were then applied to a 1·5 × 25cm column of DEAE-cellulose equilibrated with the same buffer. The column was run with the starting buffer until unbound protein and Triton X-100 eluted. The bound protein was then eluted by increasing the NaCl concentration to 0·4M. Both DEAE fractions were then concentrated using single hollow fibres.

*Immunization protocol.*—New Zealand white rabbits were obtained locally and were used to produce antisera to the DEAE-bound (DEAE 1) and unbound (DEAE 2) Triton X-100 extracts of human lung tumours. The details of immunization protocol have been published elsewhere (Veltri *et al.*, 1977). The most reactive antiserum to such extracts was used throughout this study.

Before use in immunoassays the DEAE-1 antiserum was precipitated with ammonium sulphate at 35% saturation and redissolved in one half the original volume of distilled water. The latter was dialysed against 0·01M phosphate-buffered saline (PBS) for 48 h at

4°C. After dialysis the antiserum was adsorbed with copolymerized normal human serum (Avrameas and Ternynck, 1969) at a 1:10 ratio of homogenized polymer to antiserum for 1 h at 37° and overnight at 4°C. This was followed by solid-phase immunoadsorption using normal human lung 100,000 g soluble (5·0 mg protein/ml) adsorbed on to Ultragel AcA-34 by the method of Guesdon & Avrameas (1976). A batch method using 10 ml of the immunoadsorbent to 1 ml of antiserum was adsorbed as above. Such adsorption removed all immunodiffusion reactivity to normal human serum and soluble proteins of lung extracts, but a small reactivity to Triton X-100-extracted normal lung membranes remained. The latter reactivity was removed by fluid-phase adsorption of equal volumes of antiserum and normal lung membrane Triton extracts (0·5 mg protein/ml).

*Immunodiffusion.*—Following the extensive adsorption protocol described above the antiserum to DEAE-1 of the lung-tumour Triton X-100 extract was analysed by Ouchterlony double diffusion (Veltri *et al.*, 1977).

The agarose-adsorption method of De-Carvalho (1973) was used for the adsorption analyses of adsorbed DEAE-1 antiserum. In addition to the normal Triton extracts, antiserum wells were also filled with Cohn fractions (Cohn *et al.*, 1964) of normal human serum (Pentex Labs, Kankakee, IL) normal saliva or pools of soluble extracts from other normal organs. The adsorbed antiserum was then added and diffused against DEAE-column fractions and controls to determine whether any of the adsorbents removed reactivity to the lung tumour antigens.

Identity analysis was performed by Ouchterlony double immuno-diffusion using antisera specific to known tumour markers. Antisera to CEA, AFP and $\beta$-2-microglobulin were supplied by Drs Charles Todd and Marianne Egan, Dr Robert McIntire and Dr Ralph Reisfeld respectively. These and our antiserum were cross-tested against lung-tumour Triton X-100 extracts to rule out cross reactivity. Antisera to serum proteins known to be elevated during cancer were obtained from Behring Diagnostics (Sommerville, N.J.) and were tested as above.

*Counterimmunoelectrophoresis* (CIEP).—Glass plates 8·2 cm × 10 cm were coated with 20 ml of a solution of 1% Noble agar (Difco, Detroit, MI) in 0·032M barbital-acetate buffer, pH 8·6 (Ashcavi, 1973). Paired wells 4 mm in diameter were cut in the agar at an edge-to-edge distance of 5 mm for lung TAMA 1 determinations.

For the lung TAMA-1 determinations the antiserum was carbamylated according to the method of Weeke (1968) and added to the cathodal well of each pair. Antigens and control extracts were added to the anodal wells. The plates were electrophoresed for 90 min at 125 V, 20 mA at 4°C.

*G-200 Sephadex chromatography.*—The medium grade of Sephadex G-200 (Pharmacia Fine Chemicals, Piscataway, N.J.) was prepared according to manufacturers' instructions. The running buffer was 0·01M K-phosphate (pH 7·0) containing 0·1% Na azide and 0·10% Triton X-100. The column was 1·6 × 100cm and it was prepared and run at room temperature at a flow rate of about 20 ml/h. In order to properly calibrate the column we used a series of commercially prepared markers (Pharmacia Fine Chemicals) including Blue dextran ($2 \times 10^6$ mol.-wt) ferritin (440,000) adolase (158,000) ovalbumin (43,000) ribonuclease-A (13,700) and catalase (232,000). The lung TAMA-1 was detected in column fractions after concentration by immunodiffusion with our adsorbed DEAE-I antiserum.

*Isoelectric focusing* (IEF).—The model 212 (ISCO, Lincoln, NB) 1 × 23 cm jacketed isoelectric focusing column coupled to a Model UA-5 adsorbance monitor Model 493 electrophoresis power supply, and Model 432 programmed electrophoresis pump were used for additional purification of lung TAMA-1. Linear 5–40% sucrose gradients containing 0·10% Triton X-100 and 2% Bio-Lyte ampholytes, pH 3–10 (BioRad Labs, Richmond, CA) were prepared with an ISCO Model 570 gradient marker. A 50% sucrose cushion was placed in the bottom of the IEF column before application of the gradient and samples. The ampholyte and samples were then applied to the 50% sucrose cushion with a peristaltic pump interfaced with a 3-way valve (Pharmacia, Piscataway, N.J.). The samples in 25% sucrose were applied at midpoint of the gradient, about 13 ml. The upper anodic chamber contained 1·0% phosphoric acid and the lower cathodic chamber was filled with 1·0% sodium hydroxide in 40% sucrose. The column was maintained at 4°C throughout our experiments.

All runs were made at a constant wattage of 0·42 W and the voltage never exceeded 1100 V during the average run of 43 h. Periodically the column was monitored at 280 nm using a programmed electrophoresis pump and a 1cm path flow-through cell to assess separation progress. At the end of the run the column contents were pumped through the flow-through cell for a final 280nm scan and collected in 1ml volumes and the pH of each fraction was determined. Regions showing high absorbance were pooled and the pH again determined. The pooled regions were dialysed overnight against 0·01M PBS (pH 7·2) to remove sucrose and ampholytes. Each pooled region was then tested for lung TAMA reactivity by immunodiffusion with the rabbit antisera to DEAE-1.

*Immunoelectrophoresis* (IEP).—Immunoelectrophoretic analysis of lung TAMA-1 used 0·85% Ion Agar (Oxoid, London) prepared in sodium barbital buffer, ionic strength = 0·05 and pH = 7·6. The electrophoresis step was made using 38 V and 5 mA per 1 × 3in microscope slide at 4°C for 3 h. Subsequently, rabbit anti-lung TAMA-1 was added to the trough after electrophoresis, and the slides were incubated overnight at room temperature in a humidified chamber. After this ID step, the slides were washed in 0·3M NaCl for 48 h with several changes, air dried at room temperature, stained in 0·2% amido black, and destained in 5:1:1 methanol: distilled water-acetic acid.

*Immunoperoxidase technique*.—Formalin-fixed embedded sections of a human squamous-cell carcinoma of the lung as well as formalin-fixed normal human bronchus were used to test for binding of anti-lung TAMA-1 *in situ*. The method is that described by Grandlund & Andrese (1977).

### RESULTS

### *Identification and purification of lung TAMA*

Identification of lung TAMA was made using rabbit antiserum to unbound DEAE Fraction I prepared from a Triton X-100 extract of a squamous cell carcinoma of a 65-year-old white male. The antiserum was adsorbed with pools of normal human serum, normal human lung soluble extract and Triton X-100 extracts of normal lung tissues. The antiserum was then shown to give a single precipitin band when reacted

FIG. 1.—Identification of lung TAMA-1. The adsorbed rabbit antiserum to a DEAE I eluate of a lung-tumour Triton membrane extract (TTME) (1, centre well) was tested against TTME (2, a DEAE-1 eluate of this extract) as well as normal-lung Triton membrane extract (NLTME) (3, pool) normal serum pool (NSP) (4), normal lung soluble pool (NSLP) (5), and DEAE II of TTME (6). A single precipitin line shows immunological identity between lung TAMA-1 in TTME (1) and its DEAE I (2) eluate, whereas all controls are non-reactive.

against a human lung-tumour Triton membrane extract and a DEAE I eluate of the same (Fig. 1). This antigen was termed Lung TAMA-1.

Further purification of lung TAMA-1 was as follows. Starting with a squamous-cell carcinoma Triton X-100 membrane extract containing 8·05 mg protein we obtained 2·24 mg of protein in the DEAE I unbound fraction which had lung TAMA-1 reactivity by ID (Fig. 1). The remaining protein which eluted off the column at a higher salt concentration was devoid of any such reactivity. Hence, ~72% of the total protein had been eliminated by this first purification.

One mg of the protein from the above DEAE I region was brought to 25% sucrose and added to the IEF column described above. The final 28nm scan is shown in Fig. 2; these were collected in 20 × 1ml fractions which were pooled into 6 regions designated R1–R6. The lung

FIG. 2

FIG. 2(a)

FIG. 2 and 2a.—Purification of lung TAMA-1 by isoelectric focusing (IEF). One mg of a TTME, DEAE I eluate was electrofocused in a 5–40% linear sucrose gradient containing a 3–10pH ampholyte gradient for 43 h at a constant wattage of 0·42 W. The elution profile is illustrated in Fig. 2 where the abscissa indicates the pH and absorption at 280 mn while the ordinate indicates the fractions. The 280nm absorption profile (—) has superimposed upon it the pH of each fraction (●●). ID+ indicates the pooled region (R–1) which contains lung TAMA-1. Fig. 2a illustrates that only R-1 from the IEF column contains the lung TAMA-1 found in the DEAE I starting material (C).

TAMA-1 reactivity was demonstrated by ID only in R-1 (Fig. 2a) which had a protein value of 100 μg or 10% of the loading protein concentration. The pH of R-1 was 3·0.

An additional mode of purification of lung TAMA-1 used Sephadex G-200 chromatography. We loaded 3 ml containing 600 μg protein of a DEAE I lung-tumour membrane Triton extract on to the 1·6 × 100cm column described above. The lung TAMA-1 and the calibration markers were run through this column × 3 to obtain the results shown in Fig. 3. Our antigen always eluted with about 45% of the loaded protein at a $K_{av}$ of 0·25 which extrapolates to a mol. wt of 200,000

*Immunoelectrophoresis of lung TAMA-1*

Using the IEP conditions described above we electrophoresed 20 μl of a Sephadex G-200 concentrate of lung TAMA-1 as well as 20 μl of a comparable Sephadex G-200 concentrate of normal lung Triton membrane extract (NLTME). After electrophoresis we added adsorbed antiserum specific to lung TAMA-1 to the trough and incubated the plate in a moist chamber overnight at 4°C. The final stained IEP plate is shown in Fig. 4 and demonstrates the slow cathodic mobility of lung TAMA-1 at pH 7·6. The results support the need to carbamylate our antisera before CIEP.

*Adsorption analysis by immunodiffusion*

The DEAE-1 antiserum which had been adsorbed as described above was further adsorbed by the DeCarvalho procedure with a variety of extracts, in order to

FIG. 3.—Mol.-wt determination for lung TAMA-1. A 1·6 × 100cm Sephadex G-200 column was calibrated by making 3 consecutive runs of several mol.-wt markers (●) and plotting the $K_{av}$ on the abscissa and the mol. wt (log scale) on the ordinate. Also, the averaged K (0·25) from 3 consecutive runs of a TTME, DEAE I eluate, are plotted (○) and yielded an estimated mol. wt of 200,000 for lung TAMA-1.

assess its possible origin as a normal host protein. The immunological activity of the antiserum was unaffected by these further adsorptions (Table I). Hence there was no

TABLE I.—*Adsorption analysis of the DEAE-I lung TAMA-1: Demonstration of anti-TAMA-1 antibodies after absorbtion with normal serum fractions*

| Adsorbent | Protein (mg/ml) |
|---|---|
| Cohn fractions: | |
| I | 10·0 |
| II, III | 10·0 |
| IV | 10·0 |
| IV-4 | 10·0 |
| V | 10·0 |
| VI | 10·0 |
| Norman human lung: | |
| Soluble extracts* | 7·2 |
| Triton extracts† | 5·0 |
| Normal human organs: | |
| Soluble extracts‡ | 8·0 |
| Saliva (5 × concentrated) | 3·3 |
| Human embryonic lung: (24 weeks) | |
| Triton extracts | 0·2 |
| Soluble extracts | 2·4 |
| Human embryonic lung: (26 weeks) | |
| Soluble extracts | 2·0 |

* Pool of 6 100,000*g* solubles.

† Pool of 5 Triton X-100 extracts concentrated × 5 by ultrafiltration.

‡ Pool of 100,000*g* solubles of heart, liver, lung, kidney and spleen.

FIG. 4.—Immunoelectrophoresis (IEP) of lung TAMA-1. We electrophoresed 20 μl of a lung TAMA-1-containing Sephadex G-200 fraction (1) and 20 μl of a comparable fraction of normal-lung Triton membrane extract (3). Antiserum to lung TAMA-1 was added to the trough (2) and incubated overnight at 4°C to give the results shown on the stained plate.

indication by this protocol that the new lung TAMA-1 was a normal tissue component or that it was of embryonic origin, at least in the available foetal tracts.

*Identity analysis by immunodiffusion*

Specific antisera were obtained commercially and from investigators mentioned in Materials and Methods and were cross-tested against DEAE-1 antiserum and DEAE-cellulose Peak 1. Table II illustrates that lung TAMA-1 is not CEA, AFP, or our previously described soluble lung TAA-1, 2, or 3. A precipitating antiserum to β2 microglobulin did not react with lung TAMA-1. In addition, the lung TAMA-1 did not cross-react with several serum proteins that are elevated in cancer.

TABLE II.—*Identity analysis of the DEAE-I lung TAMA*-1

| Antiserum to | Reaction with lung TAMA-1†† |
|---|---|
| CEA* | –§ |
| AFP* | – |
| β2 Microglobulin* | 0** |
| Soluble lung TAA 1,2,3† | – |
| Human colostrum‡ | 0 |
| Human serum proteins‡ | |
| ZN-α2-Glycoprotein | 0 |
| GC-Globulin | 0 |
| Fibrinogen | 0 |
| C-reactive protein | 0 |
| Serum Cholinesterase | 0 |
| IgM | – |
| IgA | 0 |
| IgG | – |
| α1-Lipoprotein | 0 |
| β-Lipoprotein | – |

* Antiserum obtained from investigators as listed in Materials and Methods.
† Goat antiserum to previously described soluble lung TAA (Veltri *et al.*, 1977).
‡ Antisera obtained from Behring Diagnostics, Sommerville, N.J.
§ Reaction of non-identity by immunodiffusion
** No reaction.
†† DEAE I fraction.

*Occurrence of lung TAMA*-1 *in various tissue extracts*

Immunoelectrophoresis showed that lung TAMA-1 had gamma electrophoretic mobility (Fig. 4). Therefore the DEAE-1 antiserum was carbamylated to obtain an anodal migration for IgG, in order to detect the antigen by CIEP. Under these conditions lung TAMA-1 migrates towards the cathode and reacts with the carbamylated antiserum that now has an anodal mobility.

Several extracts of normal lung, lung tumours and various other tumours were screened by CIEP to determine the distribution of the lung TAMA-1 in these tissues. Table III shows that lung TAMA-1 was detected in 12/15 Triton extracts of lung tumours. The antigen was not detected in the 100,000 *g* soluble or 3M KCl extracts of lung tumours or in any of the normal lung extracts tested.

A small number of Triton extracts of other tumours was available and these were also tested for lung TAMA-1 (Table III). All these extracts were negative for the antigen.

One disturbing aspect of the above data on the distribution of lung TAMA-1 was that the antigen seemed restricted to lung-tumour membrane Triton extracts. To determine whether the isolation of these antigens was unique to the Triton protocol, the following experiment was performed. The DEAE I fraction of a Triton X-100 extract was further treated by the 3M KCl and sequential sonication procedures. The samples were then tested by CIEP for residual antigen activity.

TABLE III.—*Detection of lung TAMA*-1 *in lung extracts by counterimmunoelectrophoresis* (*CIEP*)

| Tissue extract | Protein (mg/ml) | CIEP reaction* for lung TAMA-1 |
|---|---|---|
| Lung tumour: | | |
| Triton X-100 | 0·5–0·8 | 12/15† |
| 100,000 *g* soluble | 2·0–4·0 | 0/15 |
| 3M KCl | 0·4–0·7 | 0/12 |
| Breast tumour: Triton X-100 | 1·0–1·4 | 0/5 |
| Colonic tumour: Triton X-100 | 0·94, 1·4, 1·5 | 0/3 |
| Normal lung: | | |
| Triton X-100 | 0·6–0·9 | 0/7 |
| 100,000 *g* soluble | 2·7, 3·6, 7·1 | 0/3 |
| 3M KCl | 0·46, 0·72 | 0/2 |

* Adsorbed DEAE I antiserum was used to test reactivity.

† Number of + ve samples/total tested.

TABLE IV.—*Effect of* 3M *KCl and sonication on lung TAMA*-1 *reactivity*

| Sample | Protein (mg/ml) | Treatments | CIEP reaction* (immunoprecipitin band) |
|---|---|---|---|
| DEAE-1 of lung-tumour Triton pool (lung TAMA-1) | 2·4 | None | + |
| | | 3M KCl | − |
| | | Sonication | + |

* Adsorbed DEAE 1 antiserum was used to test reactivity by counterimmunoelectrophoresis.

Table IV shows that in the untreated control samples the antigens were detected as expected. 3M KCl treatment of DEAE-1 however, caused a loss of lung TAMA-1 reactivity. Sonication of the extracts did not affect the antigen.

*Immunohistochemical localization of lung TAMA*-1 in vivo

Using the indirect immunoperoxidase bridge method we were able to demonstrate a specific reaction of anti-lung TAMA-1 with formalin-embedded sections of human squamous cell carcinoma of the lung. Fig. 5 demonstrates a positive reaction for lung TAMA-1 at a 1 : 50 dilution of the rabbit-lung TAMA-1 antiserum; a negative result was obtained when normal rabbit serum was used. The positive reaction was titrated and reactivity diluted out at 1:4000. Other negative controls included testing a 1 : 50 dilution of anti-lung TAMA-1 and normal rabbit serum against normal human bronchus. These data, although preliminary, would suggest that our antiserum might be of value in a retrospective or prospective screen of lung-cancer specimens for the production of lung TAMA-1. We have confirmed these findings in at least 5 additional lung-cancer patients, using indirect immunofluorescence with the same 1 : 50 dilution of rabbit antiserum to lung TAMA-1.

### DISCUSSION

The biochemical and immunochemical evidence supporting the existence of lung TAA is extensive. Several of the antigens are soluble and detectable in a variety of histological types of human lung tumours, but most are incompletely characterized (Schlipkoter *et al.*, 1973; Viza *et al.*, 1975; Watson *et al.*, 1974, 1975; Yachi *et al.*, 1968). We recently reported on the isolation and identification of 3 soluble proteins associated with several types of lung tumours (Veltri *et al.*, 1977) but these proved to be 3 normal serum proteins produced in excess during cancer by either the tumour or the host.

Tumour-associated membrane antigens (TAMA) have been demonstrated by *in vivo* and *in vitro* immunoassays. Hollinshead *et al.* (1974, 1975) using a partially purified lung TAMA isolated by sequential low-frequency sonication, demonstrated its reactivity by skin testing. Other investigators, using more crude lung TAMA generated by 3M KCl solubilization, have demonstrated cell-mediated immune (CMI) reactivity of lung-cancer patients to such preparations. The *in vitro* CMI methods have included lymphocyte blastogenesis (Roth *et al.*, 1975; Dean *et al.*, 1978), leucocyte-migration inhibition (Boddie *et al.*, 1975; Cannon *et al.*, 1977; McCoy *et al.*, 1977) as well as microcytotoxicity (Pierce & DeVald, 1975).

We now report the isolation and identification of a membrane-bound lung antigen (lung TAMA-1) extractable from human lung squamous-cell carcinomas with Triton X-100. An antiserum produced in rabbits against a DEAE-1 elution region of a Triton X-100 lung-tumour membrane preparation was extensively adsorbed with normal tissue constituents until it yielded a single reactivity with lung-tumour Triton extracts only. This adsorbed DEAE I antiserum was subsequently used to monitor purification and characterization of lung TAMA-1.

The adsorption analysis of this antiserum by the method of DeCarvalho (1973) and identity analysis by cross-testing antisera of known specificities by Ouchterlony double diffusion revealed that lung TAMA-1 was not one of numerous constitutive host proteins we tested. Also, lung TAMA-1 could not be identified as CEA, AFP or $\beta 2$ microglobulin, or previously described soluble lung TAA 1, 2 or 3 (Veltri *et al.*, 1977).

Lung TAMA-1 demonstrated a slow gamma-type electrophoretic mobility on immunoelectrophoresis at pH 7·6. Partial purification of lung TAMA-1 was obtained on Sephadex G-200, where the antigen eluted with 45% of the protein loaded on the column. Using a calibration curve generated with known mol. wt markers, lung TAMA-1 has a mol. wt of ~200,000. By sucrose-gradient isoelectric focusing we further purified a DEAE-I region of a lung tumour membrane Triton extract and demonstrated lung TAMA-1 to have an isoelectric point of ~3·0.

Due to the cathodic electrophoretic mobility of lung TAMA-1 it became necessary to carbamylate (Weeke, 1968) the IgG rabbit antiserum to DEAE-I, in order to perform a more sensitive immunoassay, *viz.* counterimmunoelectrophoresis (CIEP). The IgG antibody altered by carbamylation to an anodal electrophoretic mobility was used to test the presence of lung TAMA-1 in a variety of lung and other tumour Triton membrane extracts. Using CIEP we confirmed the presence of our antigen in 80% of lung tumour Triton extracts, and it was not detected in similar extracts of breast or colon carcinoma.

An important observation regarding lung TAMA-1 was its apparent denaturation by treatment with 3M KCl but not by sequential low-frequency sonication, a protocol reported to isolate lung TAMA (Hollinshead *et al.*, 1974, 1975). Such data emphasizes that different modalities of membrane solubilization may yield different TAA. These data serve to suggest that lung TAMA-1 is not likely to have been present in biologically significant amounts in 3M KCl extracts tested by several researchers (Boddie *et al.*, 1975; Cannon *et al.*, 1977; Dean *et al.*, 1978; McCoy *et al.*, 1977; Pierce & DeVald, 1975; Roth *et al.*, 1975). However, other reports by Hollinshead *et al.* (1974, 1975) are more likely to have contained lung TAMA-1. Further characterization of our lung TAMA-1 will clarify its relationship to other reported lung TAMA. We attribute the isolation of this antigen to the nondenaturing and gentle mechanism by which Triton releases proteins from membranes (Helenus & Simons, 1975).

Finally preliminary suggestions of the potential value of lung TAMA-1 came from its *in vivo* localization by immunohistochemistry, with the adsorbed DEAE-1 antiserum. These data provide impetus for future retrospective and prospective immunologic studies on a variety of lung-cancer specimens.

This project was funded by a Cancer Immunodiagnosis Contract, N01-CB-43890, from the National Cancer Institute, U.S.P.H.S. The authors wish to acknowledge the skilled technical assistance rendered by JoAnn Wright and John R. McKolanis as well as the manuscript preparation performed by Ms. Suzanne Jenkins. The chairman of Otolaryngology, Dr Philip M. Sprinkle is especially acknowledged for his continued support for research in the field of cancer in his laboratories. The co-operation of the WVU departments of Pathology and Surgery also were important to the achievement of our goals

### REFERENCES

ASHCAVI, M. (1973) Counterelectrophoresis for hepatitis B antigen. In: *Manual for Hepatitis B Antigen Testing*. Philadelphia: W. B. Saunders Co. p. 113.

AVRAMEAS, S. & TERNYNCK, T. (1969) The cross-linking of proteins with glutaraldehyde and its use for the preparation of immunoadsorbents. *Immunochemistry*, **6**, 53.

BODDIE, A. W., HOLMES, E. C., ROTH, J. A. & MORTON, D. L. (1975) Inhibition of human leukocyte migration in agarose by KCl extracts of carcinoma of the lung. *Int. J. Cancer*, **15**, 823.

BRAATZ, J. A., MCINTIRE, K. R., PRINCLER, G. L., KARTRIGHT, K. H. & HEBERMAN, R. B. (1978) Purification and characterization of a human lung tumor associated antigen. *J. Natl Cancer Inst.*, **61**, 1035.

CANNON, G. B., MCCOY, J. L., DEAN, J. H., RUBIN, D. H., HERBERMAN, R. B. (1977) Direct migration inhibition (LMI) assays of lung cancer patients. *Proc. Am. Assoc. Cancer Res.*, **18**, 229.

COHN, E. G., STRONG, L. E., HUGES, W. L. & 4 others (1964) Preparation and properties of serum and plasma proteins, IV. A system for the separation into fractions of the proteins and lipoprotein components of biological tissues and fluids. *J. Am. Chem. Soc.*, **68**, 459.

DAVIES, D. A. L. (1966) Mouse histocompatibility isoantigens derived from normal and from tumor cells. *Immunology*, **11**, 115.

DEAN, J. H., JERRELS, T. R., CANNON, G. B. & 5 others (1978) Demonstration of specific cell-mediated immunity in lung cancer to autologous tissue extracts. *Int. J. Cancer*, **22**, 367.

DECARVALHO, S. (1973) Detection of neoantigens in the serum of patients with active neoplastic diseases by the absorption-immunodiffusion method.*Oncology*, **27**, 193.

FROST, M. J., ROGERS, G. T. & BASHAWE, K. D. (1975) Extraction and preliminary characterization of human bronchogenic carcinoma antigen. *Br. J. Cancer*, **31**, 279.

GRANDLUND, D. J. & ANDRESE, A. P. (1977) Detection of Epstein-Barr virus antigens with enzyme-conjugated antibody. *Int. J. Cancer*, **20**, 495.

GUESDON, J. L. & AVRAMEAS, S. (1976) Polyacrylamide-agarose beads for the preparation of effective immunoadsorbents. *J. Immunol. Methods*, **11**, 129.

HELENUS, A. & SIMONS, K. (1975) Solubilization of membranes by detergents. *Biochim., Biophys., Acta.*, **415**, 29.

HOLLINSHEAD, A. C., SETA, E., STEWART, R. H. M., RICCI, C. & Mineo, T. C. (1975) Comparison of lung cancer antigens. *Tumori*, **61**, 125.

HOLLINSHEAD, A. C., STEWART, T. H. M., & HERBERMAN, R. B. (1974) Delayed hypersensitivity reactions to soluble membrane antigens of human malignant lung cells. *J. Natl Cancer Inst.*, **52**, 327.

LOUIS, C. J., BLUNCK, J. M. & RICHMOND, L. M. (1973) Agarose-gel electrophoresis of soluble proteins from bronchial mucosa and bronchogenic carcinoma. *Oncology*, **27**, 324.

McCOY, J. L., JEROME, L. F., CANNON, G. B., WEESE, J. L. & HERBERMAN, R. B. (1977) Reactivity of lung cancer patients in leukocyte migration inhibition assays to 3-M potassium chloride extracts of fresh tumor and tissue-cultured cells derived from lung cancer. *J. Natl Cancer Inst.*, **59**, 1413.

MAXIM, P. E., WIRTZ, G. E. & WESTFALL, S. (1976) Purification of human lung tumor associated antigens. *Proc. Am. Assoc. Cancer Res.*, **17**, 148.

MELTZER, M. S., LEONARD, E. J., RAPP, H. J. & BORSOS, T. (1971) Tumor specific antigen solubilized by hypertonic potassium chloride. *J. Natl Cancer Inst.*, **47**, 703.

PIERCE, G. E. & DEVALD, B. (1975) Micrototoxicity assays of tumor immunity in patients with bronchogenic carcinoma correlated with clinical status. *Cancer Res.*, **35**, 2477.

RAZIN, S. (1972) Reconstitution of Biological Membranes. *Biochim. Biophys. Acta*, **265**, 241.

ROTH, J. A., HOLMES, E. C., BODELIE, A. W. & MORTON, D. L. (1975) Lymphocyte responses of lung cancer patients to tumor associated antigen measured by leucine incorporation. *J. Thorac. Cardiovasc. Surg.*, **70**, 613.

SCHLIPKOTER, H. W., IDEL, H., BARSOUM, A. L. & VOLLMER, U. J. (1973) Antigens characteristic for tumors in bronchial carcinoma. *Zentralbl. Bakteriol.* [*Naturwiss.*], **158**, 109.

VELTRI, R. W., MENGOLI, H. E., MAXIM, P. E. & 4 others (1977) Isolation and identification of human lung tumor-associated antigens. *Cancer Res.*, **37**, 1313.

VIZA, D., LOUVIER, M., PHILLIPS, J., BOUCHIEX, C. & GUERIN, R. A. (1975) Solubilization of an antigen associated with certain bronchial tumors. *Eur. J. Cancer*, **11**, 765.

WATSON, R. D., SMITH, A. G. & LEVY, J. G. (1974) The use of immunoadsorbent columns for the isolation of antibodies specific for antigens associated with human bronchogenic carcinoma. *Br. J. Cancer*, **29**, 183.

WATSON, R. D., SMITH, A. G. & LEVY, J. G. (1975) The detection by immunodiffusion of tumor associated antigenic components in extracts of human bronchogenic carcinoma. *Br. J. Cancer*, **32**, 300.

WEEKE, B. (1968) Cabamylated human immunoglobulins tested by electrophoresis in agarose and antibody containing agarose. *Scan. J. Clin. Lab. Invest.*, **21**, 351.

YACHI, A., MATSUURA, Y., CARPENTER, C. M. & HYDE, L. (1968) Immunochemical studies of human lung cancer antigens soluble in 50% ammonium sulfate. *J. Natl Cancer Inst.*, **40**, 663.